# The predictive capability of immunohistochemistry and DNA sequencing for determining *TP53* functional mutation status: a comparative study of 41 glioblastoma patients

**DOI:** 10.18632/oncotarget.27252

**Published:** 2019-10-22

**Authors:** Aarash K. Roshandel, Christopher M. Busch, Jennifer Van Mullekom, Joshua A. Cuoco, Cara M. Rogers, Lisa S. Apfel, Eric A. Marvin, Harald W. Sontheimer, Robyn A. Umans

**Affiliations:** ^1^ College of Agriculture and Life Sciences, Virginia Tech, Blacksburg, VA 24061, USA; ^2^ The Fralin Biomedical Research Institute at VTC, Roanoke, VA 24016, USA; ^3^ Virginia Tech School of Neuroscience, Blacksburg, VA 24061, USA; ^4^ Carilion Clinic, Division of Neurosurgery, Roanoke, VA 24014, USA; ^5^ Edward Via College of Osteopathic Medicine, Blacksburg, VA 24060, USA; ^6^ Department of Statistics (MC0439), Hutcheson Hall, Blacksburg, VA 24061, USA

**Keywords:** p53, TP53, immunohistochemistry, gene sequencing, glioblastoma multiforme

## Abstract

Tumor protein 53 (p53) regulates fundamental pathways of cellular growth and differentiation. Aberrant p53 expression in glioblastoma multiforme, a terminal brain cancer, has been associated with worse patient outcomes and decreased chemosensitivity. Therefore, correctly identifying p53 status in glioblastoma is of great clinical significance. p53 immunohistochemistry is used to detect pathological presence of the *TP53* gene product. Here, we examined the relationship between p53 immunoreactivity and *TP53* mutation status by DNA Sanger sequencing in adult glioblastoma. Of 41 histologically confirmed samples, 27 (66%) were immunopositive for a p53 mutation via immunohistochemistry. Utilizing gene sequencing, we identified only eight samples (20%) with *TP53* functional mutations and one sample with a silent mutation. Therefore, a ≥10% p53 immunohistochemistry threshold for predicting *TP53* functional mutation status in glioma is insufficient. Implementing this ≥10% threshold, we demonstrated a remarkably low positive-predictive value (30%). Furthermore, the sensitivity and specificity with ≥10% p53 immunohistochemistry to predict *TP53* functional mutation status were 100% and 42%, respectively. Our data suggests that unless reliable sequencing methodology is available for confirming *TP53* status, raising the immunoreactivity threshold would increase positive and negative predictive values as well as the specificity without changing the sensitivity of the immunohistochemistry assay.

## INTRODUCTION

Tumor protein 53 (p53) is a nuclear phosphoprotein involved in fundamental pathways of cellular growth and differentiation, including induction of apoptosis, commencement of deoxyribonucleic acid (DNA) repair, and transient arrest of the cell cycle in the G1 phase. p53 executes most of these cellular processes as a transcription factor, binding to DNA and regulating gene expression. p53 regulation of the cell cycle occurs by activating transcription of the *WAF1* gene [1]. The p21 protein is the translated product of *WAF1* and functions to inhibit cyclin-dependent kinases. This regulation ultimately causes the cell cycle to arrest in the G1 phase. Within the G1 phase, cellular DNA damage is repaired prior to each mitotic cycle, which precludes the dissemination of DNA errors and prevents tumorigenesis. As such, the gene encoding tumor protein 53 (*TP53*), is classified as a tumor suppressor gene as loss of function leads to tumor inception.

Due to its prominent role in cell cycle regulation, p53 has numerous mechanisms to restrict uncontrolled cell division from occurring. The activity of this protein is predominantly regulated at the post-translational level. Mouse double minute 2 homolog (MDM2), encoded by the *MDM2* gene, is a ubiquitin ligase that functions as an important negative regulator of p53 [2]. MDM2 binds and ubiquinates p53, resulting in protein degradation. Moreover, the turnover rate of p53 can be indirectly regulated by p14 adenosine diphosphate ribosylation factor (p14ARF). p14ARF, encoded by the *cyclin-dependent kinase inhibitor 2A* (*CDK2NA*) gene, binds and inhibits MDM2 [3]. Molecular interactions between p53, MDM2, and p14ARF fastidiously regulate the balance between the synthesis and turnover of p53, and thus, control the progression of the cell cycle.

Functional mutations in *TP53* are of considerable significance in neuro-oncology as aberrant p53 expression in glioblastoma multiforme (GBM), a terminal brain tumor, has been associated with worse patient outcomes and decreased chemosensitivity to temozolomide [4, 5]. Mutant *TP53* occurs in 30–40% of primary GBM cases, the majority of which are missense mutations occurring between exons five and eight [6]. Moreover, secondary GBM exhibits *TP53* mutation rates exceeding 90% [7]. A mutant *TP53* gene product may result in constitutive upregulation of p53 nuclear expression with potential loss of p53 function, gain of p53 function with partial conservation of wild-type protein function, or dominant negative regulation [8]. Collectively, mutant *TP53* impedes the correction of DNA errors, thus fostering gliomagenesis.

Currently, p53 immunohistochemistry (IHC) is used as a surrogate assay for the presence of mutant *TP53* in gliomas. Mutant p53 circumvents normal cellular degradation and accumulates in the nucleus, allowing aberrant p53 to be detected by IHC [8]. As mutations in *TP53* are rare in non-neoplastic brain parenchyma, neighboring tissue usually demonstrates weak nuclear staining in only a few cells [8]. Nonetheless, p53 nuclear positivity exceeding 10% in tumor cells has controversially been considered a predictor for mutant *TP53* in gliomas in prior studies [9, 10].

To our knowledge, there are currently 10 published studies investigating the correlation of p53 immunoreactivity with DNA sequencing, specifically in gliomas [7, 9–17]. Over the last 25 years, these reports have demonstrated unreliable concordance rates between p53 IHC and *TP53* mutation status ranging from 55–89% in grade I–IV gliomas. Moreover, in the same studies, the false-positive rate (the incidence of p53 IHC positivity with wild-type *TP53* presence) has ranged from 2–45%. These inconsistent results may be attributed to historically vague grading systems used in p53 IHC analysis as well as the limited sensitivity of sequencing methods (i. e., single-strand conformation polymorphism analysis) used in the early 1990’s. Nevertheless, the reliability in using p53 IHC as a surrogate to predict the mutation status of *TP53* remains a contentious topic of discussion in neuro-oncology.

Ultimately, while the standard of care for gliomas has been in existence for over a decade, there is still no cure [18]. As technologies outside of IHC have advanced, research aims to identify aberrations specific to gliomas that could be utilized as prognostic markers and potential therapeutic targets [19, 20]. Since the advent of whole genome sequencing, various groups have started to identify pathways and their associated mechanisms in glioma progression and glial cell malignancy [21, 22]. These state of the art sequencing methods provide the most reliable diagnostics as they identify the actual mutation present and are not susceptible to complications from interpretation or biology. The World Health Organization has also updated the glioma classification beyond the means of classical IHC categories to include molecular features, with *TP53* being one of these alterations [23]. Therefore, various glioma sequence analyses have now been embraced as a fundamental means to help improve diagnosis and treatment of these deadly brain tumors.

Here, we reexamine the correlation between p53 immunoreactivity and the functional mutation status of *TP53* attained by DNA Sanger sequencing in 41 adult GBM samples. The present study represents one of the largest cohorts to date, which investigates the validity of this controversial relationship in adult GBM. As such, this study supports new criteria for accurate prediction of *TP53* mutation status using p53 IHC in GBM patient samples.

## RESULTS

### Immunohistochemistry

**Figure 1:**
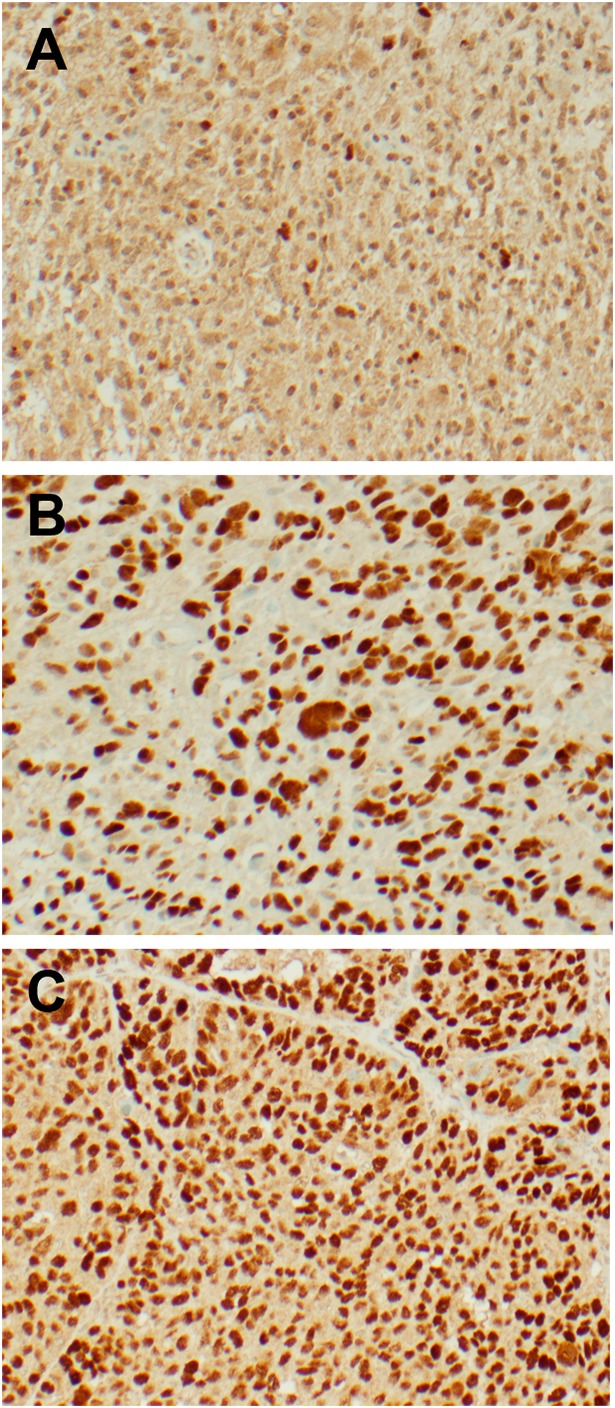
Representative IHC images from freshly resected tumor samples. p53 immunohistochemistry was performed with the Associated Regional and University Pathologists, Inc. laboratory utilizing the DO-7 antibody clone. Images were taken at 200x magnification. (**A**) Glioblastoma sample with wild-type p53 demonstrating 5% immunoreactivity. (**B**) Glioblastoma sample with mutant p53 demonstrating 80% immunoreactivity. (**C**) Ovarian serous carcinoma (positive control) demonstrating strong immunoreactivity.

Among the 41 glioma samples collected for our Carilion Glioma Bank (CGB), p53 IHC reports had values ranging from 0-90% for p53 positive nuclei. This range of percentages for each tumor sample can be found in Table 1. Forty cases demonstrated p53 immunoreactivity whereas one case had a total lack of p53 present through this method. Representative images of glioma tissue with a wild-type (panel A) and mutant (panel B) p53 status as claimed through IHC are shown in Figure 1. The clinical pathologist’s report for each tumor sample and results of additional immunohistochemistry and molecular sequencing tests can be found in Supplementary Table 1.

**Table 1:** Raw data and demographics of GBM patients

CGB patient sample #	p53 Immunoreactivity %	Mutation (Amino acid change)	EGFR amplification	R72P polymorphism	Age	Gender	Race
CGB1^*^	90	H179R	—	No	31	M	W
CGB2	5		+	No	66	M	W
CGB4	10		—	Yes	83	M	W
CGB5	25		—	Yes	63	F	W
CGB8	30		+	No	73	M	W
CGB10	5		—	Yes	66	F	W
CGB11	2		—	Yes	64	M	W
CGB12	80	R273C	+	Yes	48	M	W
CGB17	5		—	Yes	54	F	W
CGB18	5		+	No	54	F	B
CGB21	60		—	Yes	68	F	W
CGB23	1		—	Yes	79	M	W
CGB24	10		—	No	76	M	W
CGB26	5		+	No	62	F	W
CGB27	40		+	Yes	59	M	W
CGB28	10		—	No	77	F	W
CGB30	20		—	No	64	F	W
CGB33	20		—	Yes	64	F	W
CGB36	25		+	Yes	66	M	W
CGB37	20		—	No	75	F	W
CGB39	5	P108P^**^	+	No	81	F	W
CGB44	40	A158H	—	Yes	73	F	W
CGB47	1		—	No	54	M	W
CGB48	70	R273H	—	Yes	37	M	W
CGB49	10		—	No	76	M	B
CGB50	25		+	No	77	F	W
CGB51	10		+	Yes	54	M	W
CGB54	5		—	No	64	F	W
CGB55	0		—	Yes	84	M	W
CGB56	60		—	Yes	62	M	W
CGB57	25		—	Yes	65	M	W
CGB58	80	Y234D	—	Yes	80	M	W
CGB59	5		—	Yes	85	M	W
CGB60	5		—	No	54	F	W
CGB61	5		—	No	71	F	B
CGB63	90	M246T	—	Yes	31	M	H
CGB65	10		+	Yes	58	F	W
CGB66	20		—	Yes	40	F	W
CGB67	30		—	Yes	74	M	W
CGB68	75	N235D	—	Yes	63	F	W
CGB69	90	C176Y	—	Yes	65	M	W

Patients were de-identified and samples were numbered to recognize each CGB tumor sample. For all of the GBM samples used in this study, the p53 mutation statuses and EGFR amplification as deemed by IHC and demographics such as age, gender, and race are listed. Any R72P *TP53* polymorphism discovered from Sanger sequencing is also listed. Legend: ^*^secondary glioblastoma, ^**^ silent mutation, Gender: M = Male, F= Female, Race: W= White, B = Black, H= Hispanic.

### DNA sequencing

**Figure 2:**
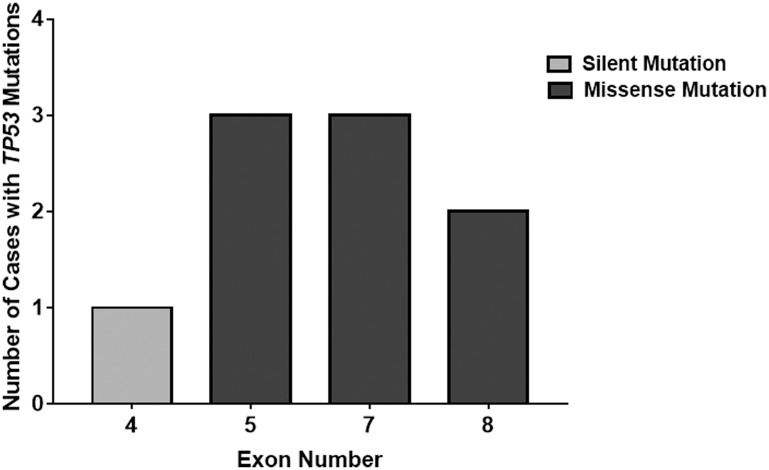
Location distribution of *TP53* mutations in freshly collected patient samples. This histogram details the mutations in different *TP53* exons discovered from Sanger sequencing analysis. All mutations were found within exons four to eight. However, all mutations in exons five to eight contained functional, missense *TP53* mutations unlike the silent mutation found in exon four.

Nine mutations were detected in 41 samples, which included eight missense mutations and one silent mutation. All mutations were found within exon four through exon eight (Figure 2). One mutation was found in exon four (c.108G>A), three mutations were found in exon five (c.473G>A, c.527G>A and c.536A>G), three mutations were from exon seven (c.700T>G, c.703A>G, c.737T>C), and two mutations were from exon eight (c.817C>T and c.818G>A). Two of the nine mutations (22%) reported in our analysis were identified in codon 273 in exon eight. The average age of patients with *TP53* mutations was 49.3 years. Six patients (67%) with *TP53* mutations were male. Eight patients (89%) were Caucasian and one patient (11%) was Hispanic. Of the samples with *TP53* mutations, two patients (22%) exhibited amplification of the extracellular growth factor receptor (EGFR) and seven patients (78%) were positive for the R72P *TP53* polymorphism. This raw data from this study is cataloged in Table 1.

### The relationship between p53 IHC staining and TP53 DNA sequencing

**Figure 3:**
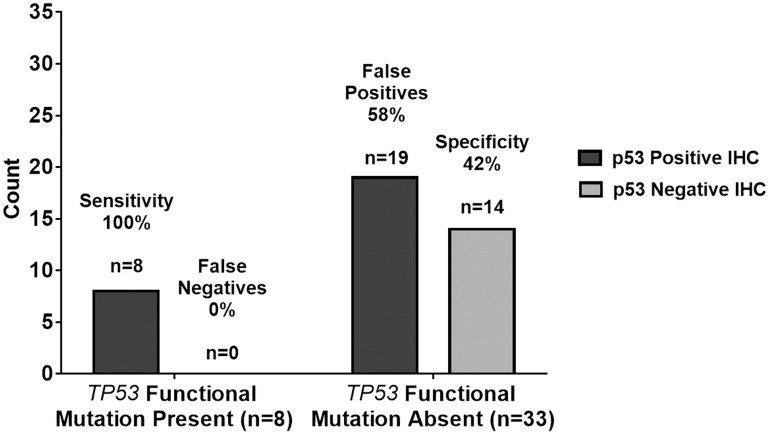
Graphical summary of *TP53* genetic sequencing results versus p53 IHC staining test results with a ≥10% threshold As summarized in tabular form, the sensitivity and specificity in using p53 IHC surrogacy as a predictor of *TP53* mutational status were 100% and 42%, respectively. The positive-predictive and negative-predictive values were 30% and 100%, respectively. The 100% sensitivity indicates the p53 IHC staining test results with a ≥10% threshold agreed with the *n* = 8 TP53 functional mutation present samples in all 8 cases resulting in *n* = 0 or 0% false negatives. The 42% specificity indicates the p53 IHC staining test results with ≥10% threshold agreed with the *n* = 33 *TP53* functional mutation absent samples in only 14 out of 33 cases resulting in *n* = 19 or 58% false positives.

We used a ≥10% immunoreactivity threshold for prediction of mutant *TP53* in relation to the current threshold used worldwide by many molecular laboratories. As such, IHC equal to or greater than 10% was considered immunopositive and IHC less than 10% was considered immunonegative. Immunopositivity specifically refers to ≥10% p53 IHC staining whereas immunoreactivity provides a specific % of p53 IHC staining. Therefore, according to this standard, all immunopositive samples are also immunoreactive but not all immunoreactive samples are all also immunopositive. Utilizing this threshold for immunopositivity, all 41 samples were evaluated as either immunopositive (27 cases, 66%) or immunonegative (14 cases, 34%). Of the 27 immunopositive samples, only eight samples (30%) harbored a *TP53* missense mutation. These eight samples exhibited p53 IHC greater than 40% (range 40–90%) (Table 1). Moreover, of the 14 immunonegative samples, one sample (7%) possessed a *TP53* mutation and a 5% p53 IHC. Since this sample contained a silent mutation, it was not considered a false negative and was treated as a true negative. The sensitivity and specificity in using p53 IHC surrogacy as a predictor of *TP53* mutational status were 100% and 42%, respectively. The positive-predictive and negative-predictive *values* were 30% and 100%, respectively. These quantities are summarized in tabular form in Table 2 and graphically in Figure 3.

**Table 2:** Contingency table summary of *TP53* genetic sequencing results versus p53 IHC staining test results for ≥ 10% staining threshold

		TP53 Genetic Sequencing Results			
		Positive for functional mutation	Negative for functional mutation	Row totals	
p53 IHC Staining Test	Positive	True Positives	False Positives	Total Staining Positives	Positive Predictive Value
	(≥10% Staining)	8	19	27	8/27
					29.6%
Results	Negative	False Negatives	True Negatives	Total Staining Negatives	Negative Predictive Value
	(<10% Staining)	0	14	14	14/14
					100%
	Column Totals	Total Mutation Positives	Total Mutation Negatives	Overall Total 41	Accuracy 22/41 53.6%
		8	33		
		Sensitivity	Specificity		
		8/8	14/33		
		100%	42.4%		

Contingency table and associated quantities with *TP53* genetic sequencing results as the standard and p53 IHC staining test results with a ≥ 10% staining threshold as the screening test. Sensitivity and specificity are calculated out of column totals representing the percentage of true positives and true negatives in reference to the standard *TP53* genetic sequencing results, respectively. Positive and negative predictive values are calculated in reference to the row totals, representing the number of *TP53* genetic sequencing results standard values predicted correctly out of the p53 IHC staining test with a ≥ 10% staining threshold positive and negative screening results, respectively. The table indicates a high percentage of false positives and poor predictive capability for p53 IHC with a ≥ 10% staining threshold.

Recall the objective of the current study was to investigate whether ≥10% immunoreactivity for p53 is a reliable measure of *TP53* mutation status in adult GBM. The summary of the results in Table 2 and Figure 3 are consistent with previous studies, which question the reliability of the p53 IHC ≥10% immunoreactivity threshold as indicated by the low positive predictive value of 30% and the number of false positives at 58%. Further statistical analysis employed Cohen’s kappa, logistic regression, and hypothesis testing on proportions used to provide evidence beyond that of the diagnostic test summary statistics of sensitivity, specificity, false positive rates, etc. to support this premise. JMP^®^ Pro Version 13 was used for all analyses. Cohen’s kappa is an agreement summary statistic that is used to characterize absolute agreement for nominal metrics such as positive and negative test results. Kappa > 0.9 implies a high level of agreement while a kappa of 0.7 to 0.9 characterizes marginal agreement. In this study, high values of kappa would indicate that IHC is a reliable predictor of *TP53* functional mutation sequencing results. Low values would indicate the opposite. The value of Cohen’s kappa = 0.1717 (*p* = 0.0990) with a 95% confidence interval of (−0.0101, 0.3542). Kappa is not significantly greater than zero as indicated by both the *p*-value and the confidence interval covering 0. This very low value of kappa that does not significantly differ from zero indicates there is low agreement between ≥10% p53 IHC and functional *TP53* mutation test results [24]. In the Discussion section, we will propose an alternative threshold for the p53 IHC test.

## DISCUSSION

GBM is a very aggressive brain cancer with a terminal prognosis. While a cure does not exist, aspirations of personalized medicine are shaping how biomedical research may translate to the clinic. Various brain tumor studies have employed genomic sequencing techniques to assess which molecular perturbations may be responsible for this fatal pathology [19–22]. The objective of the current study was to investigate whether ≥10% p53 immunoreactivity was a reliable measure of *TP53* functional mutation status in adult GBM. To achieve this aim, we assayed 41 adult GBM cases for *TP53* mutations utilizing DNA Sanger sequencing and compared these results with those obtained with p53 IHC (Table 3). In accordance with the threshold used in prior studies, p53 IHC with ≥10% positive cells was used as the cut-off value for predicting mutant *TP53*. Under these parameters, p53 IHC positivity was demonstrated in 66% (27 of 41 cases) of GBMs. However, sequencing only detected *TP53* functional mutations in 20% of our samples (8 of 41 cases). Of the 27 cases marked positive by p53 IHC (range 10–90%), DNA sequencing confirmed eight to exhibit a functional mutation in the *TP53* gene. Therefore, the positive predictive value in using a ≥10% threshold for p53 IHC positivity was only 30%.

**Table 3:** Contingency table summary of TP53 genetic sequencing results versus p53 IHC staining test results for ≥ 40% staining threshold

		TP53 Genetic Sequencing Results			
		Positive for functional mutation	Negative for functional mutation	Row totals	
p53 IHC Staining Test	Positive (≥40% Staining)	True Positives	False Positives	Total Staining Positives	Positive Predictive Value
		8	3	11	8/11
					72.7%
	Negative (<40% Staining)	False Negatives	True Negatives	Total Staining Negatives	Negative Predictive Value
		0	30	30	30/30
					100%
	Column Totals	Total Mutation Positives	Total Mutation Negatives	Overall Total 41	Accuracy 38/41 92.7%
		8	33		
		Sensitivity	Specificity		
		8/8	30/33		
		100%	90.9%		

Contingency table and associated quantities with *TP53* genetic sequencing results as the standard and p53 IHC staining test results with a ≥ 40% staining threshold as the screening test. Sensitivity and specificity are calculated out of column totals representing the percentage of true positives and true negatives in reference to the standard *TP53* genetic sequencing results, respectively. Positive and negative predictive values are calculated in reference to the row totals, representing the number of *TP53* genetic sequencing results standard values predicted correctly out of the p53 IHC staining test with a ≥ 40% staining threshold positive and negative screening results, respectively. This table based on the p53 IHC staining test with a threshold of ≥ 40% indicates a lower percentage of false positives and a higher positive predictive value in comparison to the same test based on a threshold of ≥ 10% in Table 3.

### False positives

As shown in Table 2 and Figure 3, the positive predictive value was 30% using a ≥10% threshold for p53 IHC positivity with a corresponding false positive rate of 70%. The ideal p53 screening test would have a much larger positive predictive value and a much lower false positive rate. An explanation for the high number of false-positive results in our study may be due to the specificity of the DO-7 clone used in the IHC assay. DO-7 is the most commonly used antibody clone for p53 IHC. However, the epitope recognized by DO-7 does not discriminate between wild-type and mutant p53 proteins [25]. As such, false positivity occurs in cases where wild-type p53 is overexpressed or in cases where wild-type p53 exhibits a prolonged half-life. Cellular stressors (e. g., heat, oxidative stress, irradiation, chemotherapeutic agents) are common reasons for half-life prolongation of wild-type p53 proteins [8].

### Silent mutation false negative

Interestingly, one of 14 GBM cases with less than 10% p53 IHC exhibited a mutation confirmed by Sanger sequencing. Specifically, this case (CGB39) demonstrated 5% p53 IHC staining. As such, this case did not meet the ≥10% threshold to be marked positive by p53 IHC. Ultimately this was not considered a false positive value due to the nature of the mutation observed. However, this anomaly is worth discussing further.

There are several explanations for this unusual finding observed in our study, each of which highlights an intrinsic flaw of using p53 immunoreactivity to predict *TP53* mutational status. First, the most likely explanation is that the sample had a silent mutation (P36P). It is well known that silent mutations do not alter the integrity of translated protein products. As such, translated *TP53* did not demonstrate mutational characteristics nor was it overexpressed, which impeded positive IHC staining from occurring.

Additional explanations involving protein translation and *TP53* gene regulation can cause false-negativity to occur. Nonsense, frameshift, or deletion mutations (not consisting of multiples of three nucleotides) can cause incomplete translation of p53 resulting in a truncated protein product or loss of protein expression. The resulting anomalous p53 structure may not be recognized by the DO-7 clone during p53 IHC analysis resulting in false negativity. Additionally, increased expression of MDM2, has been shown to strongly repress mutant p53 accumulation in tumors cells [26–30]. Overexpression of MDM2 has been described by several reports as a frequent molecular anomaly in GBMs [31, 32]. Moreover, the product of CDK2NA expression, p14ARF, fosters MDM2 degradation [3]. As such, overexpression of MDM2 or CDK2NA homozygous deletions can cause degradation of mutant p53 with either mechanism potentially resulting in a false-negative p53 IHC result. For example, Khatri and colleagues reported a mutant MDM2 single-nucleotide polymorphism to be more prevalent in 98 patients with GBM (54.6%) compared to 102 healthy controls (41.2%) (*p* = 0.0092) [31]. Nakamura et al., also reported CDK2NA homozygous deletions or methylation in 58% of patients with primary or secondary GBM (*n* = 50) [33]. Furthermore, overexpression of EGFR may also impact p53 IHC. Downstream in the EGFR signaling cascade, AKT phosphorylation leads to the activation of MDM2 [34, 35]. As previously discussed, high levels of MDM2 can then repress mutant p53 expression. EGFR overexpression was found in 27% (11 of 41) of our samples (Table 1). Interestingly, EGFR was overexpressed in our false negative sample. One or more of these explanations may have been a contributing factor for the false-negative result observed in the present study.

### Mutations

Our analysis found nine *TP53* mutations in nine different GBM cases. All mutations were found between exons four and eight of the *TP53* gene. One mutation was found in exon four (c.108G>A), three mutations were found in exon five (c.473G>A, c.527G>A and c.536A>G), three mutations were from exon seven (c.700T>G, c.703A>G, c.737T>C), and two mutations were from exon eight (c.817C>T and c.818G>A). Two of the nine mutations (22%) reported in our analysis were identified in the same codon, 273, in exon eight. A recent study by Shajani-Yi and colleagues demonstrated similar results, reporting 13 of 55 (24%) of *TP53* mutations identified in glioma cases to be in the 273 “hotspot residue” [36]. Furthermore, according to the IARC *TP53* Database, we report for the first time a novel missense mutation of the *TP53* gene c.473G>A in exon five.

The nine cases with sequencing confirmed mutations had an average of 69% (range 5–90%) p53 positive IHC staining. Eight of the nine cases with confirmed mutations demonstrated p53 staining of 40% or greater. Using a ≥10% threshold for p53 IHC positivity resulted in a sensitivity of 89% and false-negative rate of 11%. Comparatively, 32 cases did not exhibit a mutation when sequenced. Thirteen cases were correctly marked negative by p53 IHC yet, 19 cases were falsely deemed positive in the absence of a mutation. This resulted in a specificity of 42% and false-positive rate of 58%. Taken together with the statistical hypothesis testing in the Results section, these data challenge the reliability in utilizing a threshold of ≥10% p53 IHC positivity to predict functional *TP53* mutations in adult GBM. Figure 3 shows a summary of the predictive capabilities of the ≥10% p53 IHC for *TP53* mutations.

### A proposed new p53 IHC threshold for GBM

**Figure 4:**
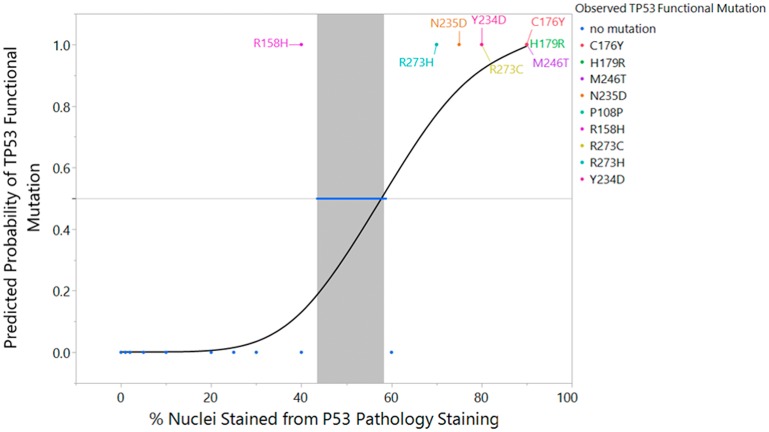
Logistic regression fit of *TP53* sequencing result versus p53 IHC % staining with inverse prediction interval and mutation label. An overlay of the actual data (0 = mutation absent, 1 = mutation present), the fitted logistic regression curve, and corresponding lower 95% confidence interval on the inverse prediction for the PS_50_ is represented in graphical form. (*p* < 0.0001, Inverse Prediction Lower 95% Confidence Interval on PS_50_ = 42.4%). Each of the eight samples and its functional *TP53* mutation is labeled appropriately within the graph.

Logistic regression was used to suggest a new, more accurate threshold for the percent of staining for p53 IHC that is predictive of a *TP53* functional mutation. In Figure 4, we see that higher values of % p53 IHC staining are associated with a positive value for a *TP53* functional mutation and lower values of % p53 IHC are associated with negative values for *TP53* functional mutation. Figure 4 includes the overlay of a logistic regression curve and corresponding lower 95% confidence interval on the inverse prediction.

The resulting model predicts a probability of a *TP53* functional mutation and provides a confidence interval for that probability. In addition, the technique can be used to provide an inverse prediction and confidence interval on the percent of staining of the p53 IHC test. The model was statistically significant (*p* < 0.0001) with an R^2^(U) value of 0.55. Please note that values of R^2^ statistics for logistic regression should not be interpreted in the same context as those for linear regression. It is uncommon to achieve values close to one. The area under the ROC curve is AUC=0.91. AUC is a measure of sensitivity and specificity. A perfect ROC curve with no false positives and no false negatives has an AUC=1. The AUC indicates good classification capability [37].

For the purposes of determining an inverse confidence interval on the percent staining for p53 IHC, we establish the concept of a PS50 or percent staining of p53 IHC at which 50% of the population are predicted to have a *TP53* functional mutation and 50% do not have a *TP53* functional mutation. The inverse prediction for the PS50 is 55.4% p53 IHC stained with a 95% lower confidence interval of 42.3% p53 IHC and superimposed on the fitted logistic curve in Figure 4. This indicates that 40% p53 IHC is a suitable threshold for accurate prediction of a *TP53* functional mutation. Note that 40% was chosen to align with the 5% or 10% intervals at which IHC is typically reported. Thus, we advocate for increasing the threshold of p53 IHC positivity in order to increase the reliability of p53 IHC surrogacy, pending future studies which corroborate this limit with additional subjects.

**Figure 5:**
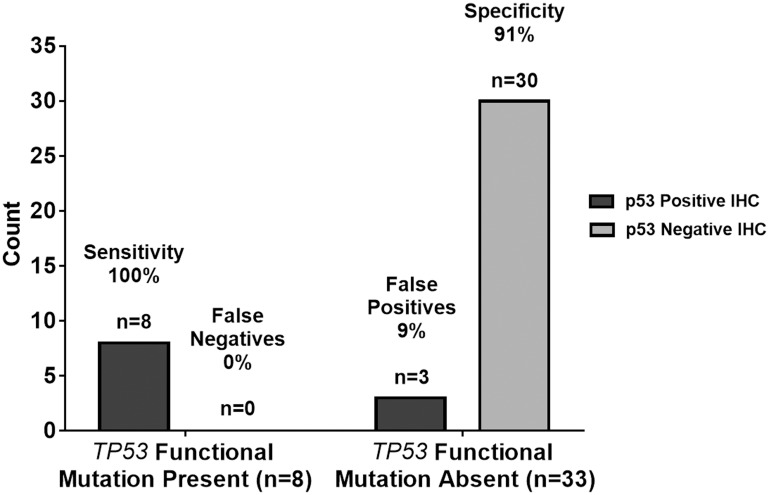
Graphical summary of *TP53* genetic sequencing results versus p53 IHC staining test results with ≥40% threshold. The 100% sensitivity indicates the p53 IHC staining test results with a ≥40% threshold agreed with the *n* = 8 *TP53* functional mutation present samples in all eight cases resulting in *n* = 0 or 0% false negatives. The 42% specificity indicates the p53 IHC staining test results with ≥40% threshold agreed with the *n* = 33 *TP53* functional mutation absent samples in 30 out of 33 cases resulting in *n* = 3 or 9% false positives.

**Figure 6:**
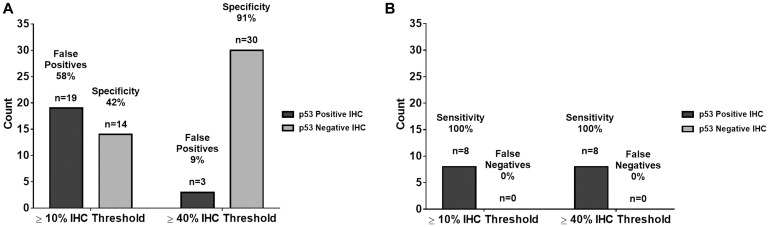
Comparison of summary statistics for ≥10% and ≥40% IHC staining thresholds. (**A**) There is no change in sensitivity and false negatives from the 10% IHC staining threshold to the 40% IHC staining threshold. (**B**) There was an improvement in specificity and false positives from the 10% IHC staining threshold to the 40% IHC staining threshold. These data show an improvement in accuracy when changing the IHC threshold.

Increasing the p53 IHC positivity threshold from ≥10% to ≥40% is validated through a comparison of the predictive capability of ≥10% and ≥40% p53 IHC thresholds versus Sanger sequencing of *TP53*. Raising the p53 IHC positivity threshold from ≥10% to ≥40% increases the positive predictive value from 30% to 73%, respectively. Under the same parameters, the negative predictive value increases from 93% to 97%. Even with a ≥40% threshold for p53 IHC positivity, the sensitivity of our study remains unchanged at 89%: eight of the nine mutations demonstrated p53 staining of ≥40%. Moreover, the specificity of our study significantly increases (*p* < 0.0001) from 42% using the ≥10% p53 IHC threshold, to 91% with a ≥40% p53 IHC cut-off. Table 3 and Figure 5 show the summary statistic results based on the ≥40% threshold as shown in Table 2 and Figure 3 in the results section for the ≥10% threshold. In Figure 6, we juxtapose the sensitivity and false negatives for the two thresholds (Figure 6A) and the specificity and false positives (Figure 6B). These figures graphically illustrate the improvement in accuracy when changing the threshold from ≥10% IHC staining to ≥40% IHC staining relative to Sanger sequencing as the standard.

In addition to investigating the correlation between p53 IHC and *TP53* functional mutation status, we also analyzed the frequency of the R72P single nucleotide polymorphism (SNP). Numerous studies have investigated the clinical manifestations of the R72P in gliomas yet the results remain inconsistent. Parhar and colleagues suggested a potential association between the R72P and predisposition to high-grade astrocytoma development in adults as well as children [38]. The small sample size of tumor DNA plus multiple ethnicities represented in the study challenged the validity of the results. Contrary to the findings by Parhar et al., numerous studies did not correlate the R72P with predisposition for glioma development [39–41]. However, in a meta-analysis encompassing eight studies (2,260 cases of glioma and 3,506 controls), Shi *et al*., found that the R72P was associated with increased susceptibility of high-grade glioma development, specifically in the European population [42]. Moreover, El Hallani et al., found an association between the R72P and an earlier age of onset in GBM [43]. We reported the prevalence of the R72P in our cohort of 41 adult GBM cases to be 61% (25 of 41 samples). All samples with a functional *TP53* mutation were positive for R72P. However, as noted in prior, the clinical ramifications of this specific SNP in GBM patients are currently unclear.

### Limitations and improvements for future studies

While this study was conducted soundly within the resource constraints available, there are some limitations that should be noted. One limitation to the current study is that we assumed molecular homogeneity of *TP53* mutations within a given tumor sample. As such, it is conceivable that mutational variability could exist within different areas of the same tumor specimen. For example, tissue extracted from the centermost portion of a tumor may exhibit a differential mutation profile from tumor abutting physiologic parenchyma. A second limitation is there is no quantification of the measurement variation of the p53 IHC test: there is inherent variability during field counting. To combat these limitations it is suggested that future studies incorporate elements of within tumor variation, laboratory to laboratory variation, within laboratory variation, and technician to technician variation. Automated image analysis may be preferable to human inspection, as this could eliminate potential bias. A larger sample size which accounts for multiple stratification factors would also aid in the corroboration of these findings.

The dataset available for this present study was limited specifically in the range of 40–60% p53 IHC staining. However, we chose a more conservative approach by selecting an IHC staining threshold of 40%. This more stringent threshold lowers the potential of accruing false negatives and positives for the p53 IHC test. We feel it is important to honor the practical, clinical implications in conjunction with the data. While there is much debate about the consequences or cost of false positives versus false negatives in the clinical setting, it can be argued that it is better to consistently attain an accurate description of cancer biomarkers (less false negatives). For example, the small molecule PRIMA-1 is a mutant p53 reactivator and currently in clinical trials for a variety of cancers [44, 45]. Accurate IHC results would provide the appropriate treatments to these patients at the expense of misidentifying some individuals of that population (more false positives) and subjecting them to unnecessary treatments that may cause a degree of morbidity.

One of our goals with this study is to prompt additional work of this type, with larger cohorts across multiple hospital networks. Such an extensive dataset could use methods such as Youden’s statistic on the predictions to quantify a more precise IHC staining threshold for widespread clinical use. Ultimately, until additional studies on larger and more diverse patient samples have been analyzed, multiple mechanisms should be used to confirm *TP53* mutational status as to not falsely label the molecular composition of a patient’s GBM.

## MATERIALS AND METHODS

### GBM tissue samples

A total of 41 histologically confirmed GBM tumor tissue samples were included in our study under the IRB approval #15-670. Samples were collected between April 2016 and May 2018 from Carilion Roanoke Memorial Hospital in Roanoke, Virginia to establish the Carilion Glioma Bank (CGB). Tissue collection occurred immediately at time of resection and samples were transported in ice cold phosphate buffered saline to a Biosafety Level-2 tissue culture room used by the Sontheimer laboratory at the Fralin Biomedical Research Institute at VTC. Tumor biopsies were rinsed again in ice cold phosphate buffered saline and dissected in glass petri dishes on ice. Samples were divided into 10–40 mg pieces and snap-frozen in cryovials. Samples remained in the -80°C freezer until DNA extraction procedures.

### DNA extraction

Each sample was weighed before DNA extraction to ensure an ideal amount of tissue could be processed. DNA was isolated per the Qiagen DNeasy Blood and Tissue Kit recommendations. Samples were re-suspended in Buffer AE for further use in polymerase chain reaction (PCR) and Sanger sequencing assays.

### PCR and Sanger sequencing

Thirteen hotspot regions, determined by the International Agency for Research on Cancer (IARC), were amplified using a slightly modified version of the IARC human *TP53* PCR 2010 protocol. These genomic regions spanned exon two through 11, including splice junction sites in order to minimize the amount of missed mutations. The PCR regions amplified, primer sequences, and programs used for each reason are detailed in Supplementary Table 2. The various PCR denaturation programs can be accessed through the IARC “Detection of TP53 mutations by direction sequencing protocol” http://p53.iarc.fr/Download/TP53_SangerSequencing_IARC.pdf. PCR products were then analyzed by gel electrophoresis in order to confirm product size and purity. Samples were purified using the Qiagen QIAquick PCR Purification Kit and then were sent for Sanger sequencing at the Biocomplexity Institute of Virginia Tech to assess for functional *TP53* mutations. Forward and reverse sequencing reactions were prepared in order to confirm results in both amplification directions. Sequencing results were aligned to the World Health Organization IARC *TP53* Database human *TP53* reference genome sequence (NC_000017.10 hg38) (http://p53.iarc.fr/TP53Sequence_NC_000017-9.aspx) in order to determine functional point mutations. For samples with a mutation, the IARC *TP53* Database codon sequence (SwissProt #P04637) (http://p53.iarc.fr/p53Sequence.aspx) was also used to determine the resulting amino acid change.

### Immunohistochemistry

Tumor samples were sent to Associated Regional and University Pathologists, Inc to determine their p53 statuses via IHC utilizing the DO-7 antibody clone and a proprietary detection system. Tissue samples were stained and their p53 IHC reactivity percentages (0–100%) were recorded. Similar to the cut-off used in prior studies, a ≥10% threshold was implemented to define immunopositivity [8].

### Statistical analyses

Statistical methods employed include summary statistics, contingency table analysis, Cohen’s kappa, and logistic regression. Confidence intervals and *p*-values are given where appropriate. JMP^®^ Pro Version 13 was used for all analyses.

## CONCLUSIONS

In the current study, our data clearly indicates that the >10% IHC threshold results in a large percentage of false positive when trying to determine *TP53* functional mutations. Therefore, this threshold does not accurately predict the *TP53* functional mutation status in adult GBM samples. With only 41 samples over the course of two years, our sample size limits our ability to definitively declare a new threshold. However, this does not hinder us from suggesting an improved threshold. A logistic regression analysis of our data indicates that increasing the threshold from 10% to 40% would increase the positive and negative predictive values as well as the specificity without impacting the sensitivity of the assay. If IHC is the preferred method for future studies, then a larger scale, systematic investigation surveying samples across hospitals and states is required to support the findings of this preliminary study for increasing the threshold from >10% to >40%.

The incidence of glioblastomas is not great enough for any one medical center to quickly and effectively determine a new IHC threshold. The lack of access to Sanger sequencing at many medical facilities will necessitate the use of IHC to determine p53 status. In addition, the cost of sending samples to third party facilities may not be feasible. Although IHC is a less accurate test for assessing *TP53* functional mutations, it is worth examining a reliable threshold of IHC staining. Further investigation may determine a more accurate threshold on a larger, more diverse sample of patients and laboratories until Sanger sequencing is more cost effective and accessible.

Ultimately, our data suggests that the field of pathology should transition as soon as feasible from IHC staining to Sanger sequencing as the method for determining *TP53* mutation status. Not only does this methodology result in determining *TP53* functional mutation with much greater accuracy and precision, it also sets the foundation for a transition into personalized medicine. By determining the exact mutation and corresponding amino acid change, we can begin to investigate the underlying effects of each mutation and affected downstream targets.

Correctly identifying p53 functional status in glioma is of paramount clinical relevance as p53 is a master regulator of tumor-initiating pathways. For example, integrins, attachments to the extracellular milieu that act as biochemical sensors for cell adhesion, are expressed in GBM patient samples, vasculature, tumor cells, and contribute to an infiltrative tumor phenotype [46, 47]. Mutant p53 cancers display gain-of-function metastasis through integrin-mediated invasion and integrin down-regulation sensitizes glioma cells to chemotherapy [48, 49]. Determining p53 status in glioma tissue would distinguish the intricate mechanisms of GBM invasion as this process harbors a relationship between p53 and integrins. Therefore, proper detection of p53 in patient samples will reveal relationships among important molecular players during tumorigenesis such as integrins and those proteins yet to be identified. Such associations will advance treatments towards unique targets so drastically needed for this group of patients.

While some studies suggest the prevalence of *TP53* mutation can vary according to GBM subtype, the current standard of care is not dependent on defining a tumor’s molecular classification [50]. Pathology laboratories that use p53 IHC immunoreactivity for p53 functional status also do not discern the GBM subtype in their reports (Supplementary Table 1). Therefore, the IHC method may not align with what has been more commonly seen from gene sequencing results and further supports the use of DNA sequencing technology to identify p53 functional status over p53 IHC immunopositivity with a single cut-point. While we argue to increase the p53 IHC cut-point score based on the most rigorous statistics performed on our available sample size, our results still substantiate the superiority of sequencing methods over traditional IHC detection. As molecular markers are more routinely and accurately identified, this will help clinicians stratify patient tumors and how they should be treated as new therapies develop. Furthermore, we can study novel therapeutics for each type of mutation identified, especially knowing what targets may be dysregulated after specific *TP53* mutations. Small molecule *TP53* mutant reactivators have been discovered and are in use in multiple clinical trials [51]. Correctly identifying the *TP53* function and status in these brain tumor specimens, could re-purpose novel therapies such as these small molecules for GBM. Therefore, the opportunity to explore the mechanisms behind *TP53* mutations in glioblastoma as well as innovative therapeutic modalities is greater when using Sanger sequencing.

## SUPPLEMENTARY MATERIALS





## Abbreviations

p53: tumor protein 53; *TP53*: tumor protein 53 gene
DNA: deoxyribonucleic acid
MDM2: mouse double minute 2 homolog
p14ARF: p14 adenosine diphosphate ribosylation factor
CDK2NA: cyclin-dependent kinase inhibitor 2A
GBM: glioblastoma multiforme
IHC: immunohistochemistry
IARC: International Agency for Research on Cancer
PCR: polymerase chain reaction
EGFR: extracellular growth factor receptor
SNP: single nucleotide polymorphism
CGB: Carilion Glioma Bank
