# Supplementary material for: The predictive capability of immunohistochemistry and DNA sequencing for determining TP53 functional mutation status: a comparative study of 41 glioblastoma patients
Source: Oncotarget. 2019 Oct 22;10(58):6204–18. doi: 10.18632/oncotarget.27252 (PMC6817445; doi:10.18632/oncotarget.27252)
Supplement: Supplementary file 1 [file oncotarget-10-6204-s001.pdf]

The predictive capability of immunohistochemistry and DNA sequencing for determining *TP53* functional mutation status: a comparative study of 41 glioblastoma patients

SUPPLEMENTARY MATERIALS

Supplementary Table 1: Clinical pathology reports for GBM patient samples. See Supplementary Table 1

Supplementary Table 2: List of primers used for sanger sequencing experiments

Region	Forward sequence	Reverse sequence	Product length	PCR program per IARC protocol, 2010 update
Exons 2–3	tctcatgctggatccccact	agtcagaggaccaggtcctc	344bp	B
Exon 4.1	tgctcttttcacccatctac	atacggccaggcattgaagt	353bp	B
Exon 4.2	tgaggacctggtcctctgac	agaggaatcccaaagttcca	413bp	B
Exon 5	ttcaactctgtctccttcct	cagccctgtcgtctctccag	248bp	B
Exon 6	gcctctgattcctcactgat	ttaacccctcctcccagaga	181bp	B
Exons 5–6	tgttcacttgtgccctgact	ttaacccctcctcccagaga	467bp	B
Exon 7.1	cttgccacaggtctccccaa	aggggtcagaggcaagcaga	237bp	C
Exon 7.2	aggcgcactggcctcatctt	tgtgcagggtggcaagtggc	177bp	B
Exon 8	ttccttactgcctcttgctt	aggcataactgcacccttgg	231bp	B
Exons 8-9	ttgggagtagatggagcct	agtgttagactggaaacttt	445bp	B
Exon 9	gacaagaagcggtggag	cggcattttgagtgttagac	215bp	E
Exon 10	caattgtaacttgaaccatc	ggatgagaatggaatcctat	260bp	D
Exon 11	agaccctctcactcatgtga	tgacgcacacctattgcaag	245bp	B
