# Supplementary material for: The predictive capability of immunohistochemistry and DNA sequencing for determining TP53 functional mutation status: a comparative study of 41 glioblastoma patients
Source: Oncotarget. 2019 Oct 22;10(58):6204–18. doi: 10.18632/oncotarget.27252 (PMC6817445; doi:10.18632/oncotarget.27252)
Supplement: Supplementary file 2 [file oncotarget-10-6204-s002.docx]

**Supplementary Table 1: Clinical pathology reports for GBM patient samples**

| **CGB Patient Sample #** | **p53 immunoreactivity** | **EGFR Amplification** | **MGMT**  **Status** | **IDH Wild-Type**  **versus**  **R132 Mutant** | **Pathologist Description** |
| --- | --- | --- | --- | --- | --- |
| CGB1 | 90% | Not Amplified | Methylated | Mutant | Sections of the brain material show hypercellular proliferation of cells with cytologic atypia. There is vascular proliferation and focal necrosis. The findings are consistent with glioblastoma. |
| CGB2 | 5% | Amplified | Not Methylated | Wild-Type | A.  A section of the craniotomy material shows a proliferation of predominantly round to stellate cells without significant cytologic atypia, necrosis, or significant vascular proliferation. Focally there is increased cellularity and cytologic typical with mitotic figures to suggest a higher grade process.  B.  A section of the additional craniotomy material shows features similar to that seen in part A, however, there is increased cellularity with focal necrosis and more cytologic atypia and vascular proliferation. The tumor cells express GFAP. No significant expression is seen in the tumor cells for Keratin CAM5.2, CD45 or Melan A. The findings are compatible with a glioblastoma. |
| CGB4 | 10% | Not Amplified | Methylated | Wild-Type | A.  Section A shows glial tissue which is hypercellular and shows atypia of the constituent cells.  There is increased vascularity. There are areas of necrosis.  B.  Sections B are essentially similar. The following immunostains are performed in terms of the neoplastic cells: GFAP - positive, Melan A - negative, Ki-67 - 6% cells positive. |
| CGB5 | 25% | Not Amplified | Not Methylated | N/A | A. Levels of the occipital mass material show fragments of necrotic focally hypercellular tissue with neuropil background.  B. Sections of the additional left occipital mass material show a proliferation of noncohesive cells in a neuropil background. Tumor cells express GFAP.  No significant expression is seen with Melan-A, EMA, CD45, or keratin CAM 5.2. There is necrosis and vascular proliferation. The findings are consistent with a glioblastoma. |
| CGB8 | 30% | Amplified | Methylated | Wild-Type | A. Levels of the brain tumor material show a hypercellular proliferation of cells with cytologic atypia. There is vascular proliferation and necrosis. The findings are compatible with glioblastoma.  B. Sections of the additional brain tumor material shows histologic features as those seen in part A. Additionally tumor cells show expression of GFAP. No significant expression is seen with CD45, Melan-A, or keratin CAM 5.2. The findings are consistent with glioblastoma. |
| CGB10 | 5% | Not Amplified | Not Methylated | Wild-Type | The histologic shows a tumor consisting of large atypical cells in a fibrillary matrix. Mitotic figures are readily appreciated. There is some palisading necrosis. |
| CGB11 | 2% | Not Amplified | Not Methylated | Wild-Type | A. Sections of the left temporal brain material show a hypercellular proliferation of discohesive cells in a neuropil background. There is vascular proliferation. The findings are compatible with a glioblastoma.  B. Sections of the additional left temporal brain material show features similar to that seen in part A.  There is also focal necrosis. The findings are consistent with glioblastoma. |
| CGB12 | 80% | Amplified | Methylated | Mutant | A. A section of the left brain mass material shows a necrotic hypercellular proliferation of discohesive cells with a neuropil background.  The findings are compatible with glioblastoma.   B. Sections of the additional left frontal brain material show features similar to that seen in part A. Additionally there is glomeruloid vascular proliferation.  The findings are compatible with glioblastoma. Immunohistochemical stains show the tumor cells to express GFAP.  No significant expression seen with CAM5.2 or CD45 in the malignant cells. |
| CGB17 | 5% | Not Amplified | Not Methylated | Wild-Type | A. A section of the right parietal lesion material shows necrosis with focal residual areas of cellular glial areas. The findings are compatible with glioblastoma. B. Sections of the additional right parietal lesion show a cellular glial proliferation with neuropil background. There is necrosis and vascular proliferation. The findings are consistent with glioblastoma.   C. Sections of the additional parietal lesion material show features similar to those seen in parts A and B consistent with glioblastoma. |
| CGB18 | 5% | Amplified | Methylated | Wild-Type | A. A section of the brain tumor material shows a glial proliferation with increased cellularity and necrosis with areas of vascular proliferation compatible with glioblastoma.   B. Sections of the additional brain material show a hypercellular glial lesion with vascular proliferation and necrosis consistent with glioblastoma.   C. Levels of the additional glial material show features similar to that seen in parts A and B consistent with glioblastoma. |
| CGB21 | 60% | - | N/A | Wild-Type | A,B. Sections are examined and culminate in the above diagnosis.  The following immunostains are performed and reported on the tumor cells as seen in block B1: GFAP is positive and keratin AE1/AE3 is negative. |
| CGB23 | 1% | Not Amplified | Not Methylated | Wild-Type | A. The histologic section shows a glial neoplasm, high grade, demonstrating palisading necrosis. |
| CGB24 | 10% | Not Amplified | Methylated | Wild-Type | A-B. The histologic sections labeled A, B1 through B5, are studied and culminate in the diagnosis of glioblastoma. |
| CGB26 | 5% | Amplified | Methylated | Wild-Type | A. Levels of the biopsy material from the right parietal brain show a proliferation comprised of increased glial cells. Overt necrosis and vascular proliferation is not identified. The findings are consistent with glioma.  B. Sections of the additional right parietal brain tumor material shows a hypercellular lesion comprised of dyscohesive cells in a neuropil background.  Necrosis and vascular proliferation are seen. The tumor cells express GFAP.  No significant expression is seen with keratin CAM 5.2.  The findings are consistent with a glioblastoma. |
| CGB27 | 40% | Amplified | Not Methylated | Wild-Type | A. A section of the left brain tumor material shows a proliferation of non-cohesive cells in a neuropil background. There is necrosis and vascular proliferation. The findings are consistent with glioblastoma.  B. Sections of the material from part B show features similar to those seen in part A.  Additionally, the tumor cells show expression of GFAP. No significant expression is seen with keratin CAM5.2. The findings are consistent with glioblastoma. |
| CGB28 | 10% | Not Amplified | Not Methylated | Wild-Type | The histologic section shows tissue with a malignant tumor in which atypical cells lie within a fibrillary matrix. There is necrosis.   The following immunostains are performed and interpreted in terms of the neoplastic cells: GFAP – positive, CD45 – negative. |
| CGB30 | 20% | Not Amplified | Methylated | Wild-Type | A. Sections of the left frontal tumor material show a hypercellular proliferation of atypical cells with increased nuclear cytoplasmic ratio. There is vascular proliferation and palisading necrosis. The findings are compatible with glioblastoma.  B. Sections of the additional left frontal brain material show features similar to that in part A. Additionally, the tumor cells express GFAP.  No significant expression with keratin CAM5.2 is seen. The findings are consistent with glioblastoma. |
| CGB33 | 20% | Not Amplified | Not Methylated | Wild-Type | A. A section of the right frontal lobe material shows a necrotic mass comprised of atypical discohesive of neuropil background. Necrosis is present. Vacular proliferation is bserved. The findings are consistent with glioblastoma.   B. Additional sections of the right frontal lobe material show features similar to that seen in part A.  Immunohistochemical stains are performed (B4) for GFAP and keratin CAM 5.2 to evaluate the atypical cells. These cells show expression of GFAP but not CAM 5.2, compatible with glioblastoma. |
| CGB36 | 25% | Amplified | Not Methylated | Wild-Type | A-B. The histologic sections can be summarized as showing a high-grade glial neoplasm with frequent mitotic figures and palisading necrosis.  CD44 immunostain (prognostic factor) performed is positive. |
| CGB37 | 20% | Not Amplified | Not Methylated | Wild-Type | A. Levels of the left parietal brain tumor material show a hypercellular proliferation of noncohesive cells with marked cytologic atypia. There is vascular proliferation and necrosis. The findings are consistent with glioblastoma.   B. Levels of the additional left frontal parietal material show features similar to that seen in part A.  Additionally, immunohistochemical stains are performed to characterize these cells and show the cells to express GFAP.  No significant expression seen with CAM5.2, CD45 or Melan-A.  The findings are compatible with glioblastoma. |
| CGB39 | 5% | Amplified | Not Methylated | Wild-Type | A. Sections of material from the right brain shows a hypercellular neoplasm comprised of discohesive cells in a neuropil background. There is vascular proliferation and necrosis.  Findings consistent with glioblastoma.   B. A section of the additional right brain material shows features similar to that seen in part A. Additional immunohistochemical stains are performed to characterize the tumor cells which express GFAP but not keratin CAM5.2, Melan-A or CD45. The findings are compatible with glioblastoma. |
| CGB44 | 40% | Not Amplified | Not Methylated | Wild-Type | The histologic sections are studied and culminate in the diagnosis of glioblastoma A CD44 immunostain (prognostic factor) shows positive membranous staining. |
| CGB47 | 1% | Not Amplified | Not Methylated | N/A | A. Levels of the right temporal brain mass material show a hypercellular proliferation of atypical noncohesive cells with neuropil background. There is vascular proliferation and necrosis. The findings are compatible with a glioblastoma.   B. Sections of the additional right temporal brain mass material show features similar to that seen in part A. Immunohistochemical stains are performed to characterize the cells. These cells express GFAP. No significant expression is seen. These cells express GFAP. No significant expression is seen in these cells for CD45, keratin AE1/AE3 or Melan  A. The findings are consistent with glioblastoma. An additional immunostain is performed (CD44 prognostic factor).  The tumor is positive. |
| CGB48 | 70% | Not Amplified | Not Methylated | Mutant | A. A section of the left frontal brain tumor material shows a hypercellular proliferation of atypical cells with neuropil background over vascular proliferation and necrosis not seen.  B. Sections of the additional left frontal brain material show features similar to that in part A. Additionally, there is necrosis and focal vascular proliferation. Immunohistochemical stains are performed to characterize these cells and show the atypical cells to express GFAP. Significant expression is not seen with CD45, melan-A or CAM5.2. The findings are consistent with glioblastoma. CD44 immunostain (prognostic factor) is positive. |
| CGB49 | 10% | Not Amplified | Not Methylated | Wild-Type | The histologic sections culminate in the diagnosis of glioblastoma. A CD44 immunostain (prognostic factor) is positive. |
| CGB50 | 25% | Amplified | Not Methylated | Wild-Type | A-C. The histologic sections culminate in the diagnosis of glioblastoma. A CD44 IHC (prognostic factor) is positive. |
| CGB51 | 10% | Amplified | Methylated | Wild-Type | A-D. The histologic sections culminate in the diagnosis of glioblastoma. A CD44 immunostain (prognostic factor) is positive. |
| CGB54 | 5% | Not Amplified | Not Methylated | Wild-Type | A, B. The histologic sections labeled A and B show a cellular glial type proliferation in which there are areas of necrosis, some palisading, as well as some vascular proliferation. The following immunostains are performed on block B to further assess the tumor cells: GFAP - positive, keratin AE1/AE3 negative, Ki-67 hot spots at 5%. |
| CGB55 | 0% | Not Amplified | Not Methylated | Wild-Type | A. The histologic section AFS1 shows white matter with at most a slight increase in glial cells.The frozen section slide is reviewed and does reveal a few more atypical glial cells.   B. The histologic section shows brain tissue with increased cellularity due to increased glial cells with gemistocytic appearance as well as smaller cells with less cytoplasm.  C. Similar to B. There is vascular proliferation and necrosis. The following immunostains are performed and interpreted to help elucidate the nature of the neoplastic cells: GFAP - positive in both large and small neoplastic cells, keratin AE1/AE3 - negative, CAM 5.2 -negative, CD45 - negative and Ki-67 - hot spots at about 20% of tumor cells. A CD44 immunostain (prognostic factor) is positive. |
| CGB56 | 60% | Not Amplified | Not Methylated | N/A | Sections of the brain tumor show fragments of markedly hypercellular brain parenchyma infiltrated by a glioblastoma composed of anaplastic fibrillary astrocytes with scattered mitoses, endothelial proliferation, and areas of necrosis. CD44 IHC positive. |
| CGB57 | 25% | Not Amplified | Methylated | Wild-Type | Sections show fragments of hypercellular brain parenchyma infiltrated by a glioblastoma composed of anaplastic fibrillary astrocytes with mitoses, endothelial proliferation, and areas of necrosis.  There is pseudopalisading of the neoplastic cells adjacent to some areas of necrosis.  CD44 IHC positive. |
| CGB58 | 80% | Not Amplified | Methylated | Wild-Type | A,B. Sections of both right parietal lobe specimens show markedly hypercellular brain parenchyma infiltrated by a glioblastoma composed of anaplastic fibrillary astrocytes with mitoses, endothelial proliferation, and areas of necrosis. A CD44 by immunohistochemistry is positive. |
| CGB59 | 5% | Not Amplified | Not Methylated | N/A | A, B. Sections of the left temporal lobe biopsy and resection show markedly hypercellular brain parenchyma infiltrated by a glioblastoma composed of anaplastic fibrillary athrocytes with mitoses, endothelial proliferation, and extensive areas of necrosis. The tumor forms an infiltrating border with adjacent gray and white matter. A CD44 immunostain is performed (prognostic factor), the tumor cells are positive. |
| CGB60 | 5% | Not Amplified | Methylated | N/A | A,B.  Sections of both right parietal-occipital lobe specimens show fragments of hypercellular cerebral cortical gray and white matter infiltrated by a glioblastoma composed of anaplastic fibrillary astrocytes with mitoses, endothelial proliferation and areas of necrosis. There is focal pseudopalisading adjacent to some areas of necrosis. The tumor forms an infiltrating border with the adjacent brain parenchyma with satellitosis in the gray matter. CD44 immunostain (prognostic factor) is positive. |
| CGB61 | 5% | Not Amplified | Not Methylated | Wild-Type | A,B.  Sections of the right insular mass show fragments of hypercellular cerebrocortical gray and white matter infiltrated by a glioblastoma composed of anaplastic fibrillary astrocytes with scattered mitoses, endothelial proliferation and areas of coagulative and fibrinoid necrosis. Focally, the tumor forms microcystic spaces. The tumor forms an infiltrating border with the adjacent brain parenchyma.  Many blood vessels in the tumor matrix and the adjacent parenchyma have ectatic or hyalinized walls, consistent with a radiation-induced change. |
| CGB63 | 90% | Not Amplified | Methylated | Mutant | A, B.  Sections of both left temporal lobe specimens show fragments of markedly hypercellular cerebrocortical gray and white matter infiltrated by a glioblastoma composed of pleomorphic, anaplastic fibrillary astrocytes with frequent mitoses, endothelial proliferation, and areas of necrosis.  The tumor forms an infiltrating border with the adjacent brain parenchyma with satellitosis in the gray matter. |
| CGB65 | 10% | Amplified | Methylated | Wild-Type | A,B,C.  Sections of all of the right temporal lobe specimens show fragments of markedly hypercellular brain parenchyma infiltrated by a glioblastoma composed of anaplastic fibrillary astrocytes with frequent mitoses, microvascular proliferation and areas of necrosis. There is focal pseudopalisading of the neoplastic cells adjacent to some areas of necrosis. The tumor forms an infiltrating border with the adjacent brain parenchyma with satellitosis in the gray matter. Focally, the tumor shows microcystic architecture. Rare microcalcifications are seen in the tumor matrix. |
| CGB66 | 20% | Not Amplified | Not Methylated | Wild-Type | A,B,C. Sections of all of the left parietal lobe specimens show fragments of marked hypercellular brain parenchyma infiltrated by a glioblastoma composed of anaplastic fibrillary astrocytes with scattered mitoses, microvascular proliferation and areas of necrosis. There is focal pseudopalisading of the neoplastic cells adjacent to areas of necrosis.  In some areas of the tumor and necrotic debris, there is a brisk acute inflammatory infiltrate with abscess formation.  The tumor cells stain strongly with an antibody to the glial fibrillary acidic protein (GFAP) but not with an antibody to keratins (CAM5.2). |
| CGB67 | 30% | Not Amplified | Not Methylated | N/A | Sections of both right parietal lobe specimens show markedly hypercellular gray and white matter infiltrate by a glioblastoma composed of anaplastic fibrillary astrocytes with mitoses, endothelial proliferation, and extensive areas of necrosis. The tumor forms an infiltrating border with the adjacent brain parenchyma. |
| CGB68 | 75% | Not Amplified | Methylated | Wild-Type | A. Section A shows a malignant tumor consisting of small cells with high mitotic activity and necrosis.  B. Section B is similar but in addition shows a more conventional glial tumor showing marked nuclear atypia. GFAP is positive in the cells that show more glial differentiation. The small cell component is negative. Keratin AE1/AE3 - negative, CAM 5.2 - negative, CD45 - negative, CD44-positive and TTF-negative.  C.  Section C shows largely necrotic tumor. |
| CGB69 | 90% | Not Amplified | Not Methylated | Wild-Type | The histologic sections culminate in the diagnosis of glioblastoma. In addition, the following immunostains (with working controls) are performed to help discern cell type: GFAP -focally positive, keratin AE1/AE3 - negative, CD45 - negative, CD20 - negative, SOX10 - stains infrequent cells. |

Legend: N/A: undetermined result.
